# Preparative Separation and Purification of the Total Flavonoids in *Scorzonera austriaca* with Macroporous Resins

**DOI:** 10.3390/molecules21060768

**Published:** 2016-06-13

**Authors:** Yang Xie, Qiu-Shi Guo, Guang-Shu Wang

**Affiliations:** 1 Department of Pharmacognosy, School of Pharmaceutical Sciences, Jilin University, Changchun 130021, China; 13514486691@163.com; 2 Department of Pharmacy, the Second Part of the First Hospital, Jilin University, Changchun 130021, China; guoqiushi126@126.com

**Keywords:** *Scorzonera austriaca*, flavonoids, macroporous resins

## Abstract

The use of macroporous resins for the separation and purification of total flavonoids to obtain high-purity total flavonoids from *Scorzonera austriaca* was studied. The optimal conditions for separation and purification of total flavonoids in *S. austriaca* with macroporous resins were as follows: D4020 resin columns were loaded with crude flavonoid extract solution, and after reaching adsorptive saturation, the columns were eluted successively with 5 bed volumes (BV) of water, 5 BV of 5% (*v*/*v*) aqueous ethanol and 5 BV of 30% (*v*/*v*) aqueous ethanol at an elute flow rate of 2 BV·h^−1^. Total flavonoids were obtained from the 30% aqueous ethanol eluate by vacuum distillation recovery. The content of flavonoid compounds in the total flavonoids was 93.5%, which represents an improvement by about 150%. In addition, five flavonoid compounds in the product were identified as 2″-*O*-β-d-xylopyranosyl isoorientin, 6-*C*-α-l-arabipyranosyl orientin, orientin, isoorientin and vitexin by LC-ESI-MS analysis and internal standard methods. The results in this study could represent a method for the large-scale production of total flavonoids from *S. austriaca*.

## 1. Introduction

*Scorzonera austriaca* Wild, a perennial herb of the *Compositae*, is widely distributed in the northeast and northwest regions in China, being especially abundant in Jilin Province. It has been widely used as a traditional herbal medicine for curing fevers, carbuncles and mastitis [1]. We have proved that the total flavonoids from *S. austriaca* have hepatoprotective effects and inhibitory effects on hepatitis B virus [2,3,4], and thus the total flavonoids from *S. austriaca* have been selected as a drug candidate for treating hepatitis B by the National Important Special Foundation of the New Drug Development of China (No. 2013ZX09103002-007). Thus, an efficient method for the separation of the total flavonoids from *S. austriaca* is needed.

To date many methods for flavonoid purification have been developed. These include high speed counter-current chromatography [5], solvent extraction, agarose gel media [6], supercritical carbon dioxide extraction [7], aqueous two-phase flotation and molecule imprinted solid phase extraction [8,9]. However, some of these methods suffer from the shortcoming of needing specific costly instruments, consuming a large amount of organic solvents, low throughput, or being unsuitable on an industrial scale. Alternatively, macroporous resin adsorption technology is an efficient method to prepare bioactive constituents with many advantages, such as a large specific surface area, less consumption of organic solvents, low cost, good mechanical properties and easy regeneration, and is favorable for industrial applications [10]. Recently, separation methods based on macroporous resins have been applied more and more in laboratories and related industries for obtaining bioactive natural products from *Olea europaea* [11], *Rabdosia serra* [12], *Dryopteris fragran*s [13], pigeon pea roots [14], *Siraitia grosvenorii* [15], and *Canarium album* [16].

However, there are no reports on using macroporous resins to separate the total flavonoids from *S. austriaca* till now, so the aim of this study was to develop an efficient method for the preparative separation and purification of total flavonoids from *S. austriaca* with the optimal resin and separation conditions. The information in this study is significant in the selection of adsorption resins and for separation and purification of total flavonoids from *S. austriaca* in particular or other herbal materials in general.

## 2. Results and Discussion

The following equations were used to quantify the capacities of adsorption and desorption as well as the desorption ratio:

Q_a_ = (C_0_ − C_e_)V_i_ / W (1)

Q_d_ = C_d_V_d_ / W (2)

R_d_ (%) = Q_d_ / Q_a_ × 100% (3)

where Q_a_ is the adsorption capacity at adsorption equilibrium (mg**·**g^−1^ dry resin); Q_d_ is the desorption capacity after adsorption equilibrium (mg**·**g^−1^ dry resin); R_d_ is the desorption ratio (%); C_0_ and C_e_ are the initial and equilibrium concentrations of total flavonoids in the solution, respectively (mg**·**mL^−1^ ); C_d_ is the concentration of total flavonoids in the desorption solution (mg**·**mL^−1^ ); V_i_ is the volume of the initial sample solution (mL); V_d_ is the volume of the desorption solution (mL); W is the weight of resin (g). 

### 2.1. Screening of Macroporous Resin 

**Figure 1:**
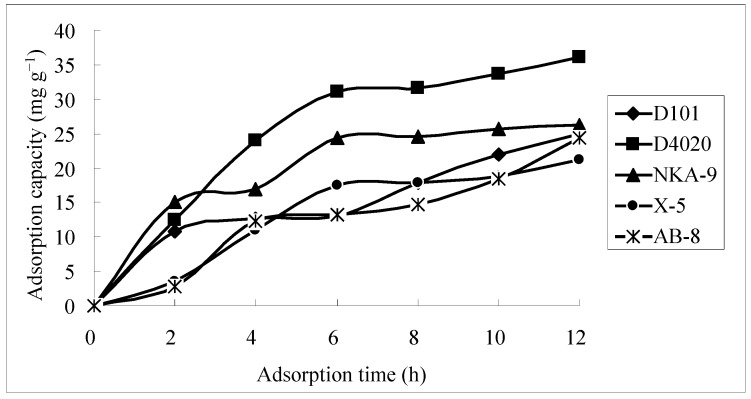
Adsorption kinetics curves for total flavonoids on different resins.

A 1.40 mg**·**mL^−1^ crude flavonoid extract solutionfrom *Scorzonera austriaca* was used as the test sample. Adsorption and desorption properties of five macroporous resins for total flavonoids were investigated. The results, shown in Table 1, indicated that among the five resins, the D4020 resin possessed a comparatively high adsorption capacity Q_a_ (38.7 mg**·**g^−1^), desorption capacity Q_d_ (34.8 mg**·**g^−1^) and desorption ratio (90.1%). The adsorption kinetics curves for total flavonoids on the five different resins were also obtained (Figure 1). From what can be seen in Figure 1, for all the five different resins, the adsorption capacity of total flavonoids increased with the adsorption time, increasing rapidly in the first 4 h and reaching equilibrium at about 6 h. For total flavonoids in the crude flavonoid extracts, all the five different resins have about the same rate of adsorption. In the comprehensive consideration of the adsorption capacity, desorption capacity, ratio of desorption and rate of adsorption, D4020 resin was fit to separate and purify total flavonoids from the crude flavonoid extracts, and in the following study, D4020 resin was chosen for further experiments. 

### 2.2. Maximum Adsorption Capacity of the Selected Resin 

**Figure 2:**
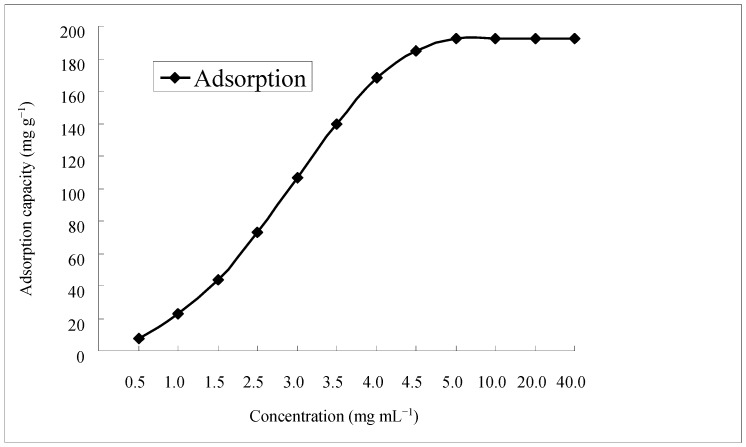
Equilibrium adsorption curve for total flavonoids in the crude flavonoid extracts on D4020 resin.

The maximum adsorption capacity of D4020 resin for total flavonoids was obtained through the equilibrium adsorption curve at the different initial concentrations of the crude flavonoid extracts, 0.5, 1.0, 1.5, 2.5, 3.0, 3.5, 4.0, 4.5, 5.0, 10.0, 20.0 and 40.0 mg**·**mL^−1^, respectively. As shown in Figure 2, the adsorption capacity of total flavonoids increased with the initial concentration, and reached the saturation plateau when the initial concentration of the crude flavonoid extracts was 10.00 mg**·**mL^−1^ and the maximum adsorption capacity was 192.1 mg**·**g^−1^, which indicated that in order to get maximum adsorption capacity, the initial concentration of the crude flavonoid extracts should be equal to or greater than 10.00 mg**·**mL^−1^. In the following dynamic adsorption and desorption study, the initial concentration of crude flavonoid extracts was chosen as 20.0 mg**·**mL^−1^. 

### 2.3. Effect of Ethanol Concentration on Ratio of Desorption in Static Desorption 

**Figure 3:**
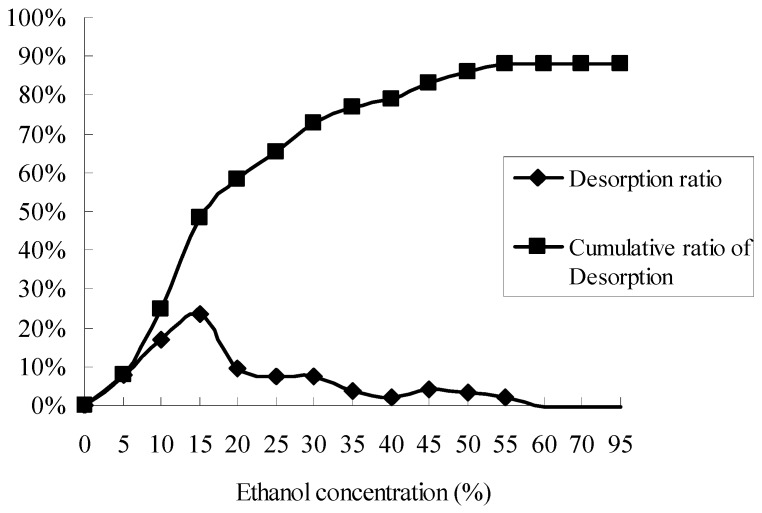
Ratios of desorption and cumulative ratios of desorption for total flavonoids on D402 resin with ethanol-water solutions of different concentrations.

In order to choose proper desorption solutions, a sample was desorbed successively with ethanol-water (5%, 10%, 15%, 20%, 25%, 30%, 35%, 40%, 45%, 50%, 55%, 60%, 70%, 95%, *v*/*v*) solutions, and the results were showed in Figure 3. At the range of ethanol concentrations from 5% to 30%, the ratios of desorption were higher than other concentrations, and more than 80% of total flavonoids were desorbed as the ethanol concentration reached 40%. Thus for the principle of efficiency and economy, the ethanol-water solution (40%, *v*/*v*) was selected as the desorption solution in investigating the elute flow rates, and the ethanol-water solutions (5%, 10%, 15%, 20%, 25%, 30%, 35%, 40%, 60%, *v*/*v*) were selected as the desorption solutions of the gradient elution in the dynamic desorption experiments.

### 2.4. Desorption Flow Rate in Dynamic Desorption 

**Figure 4:**
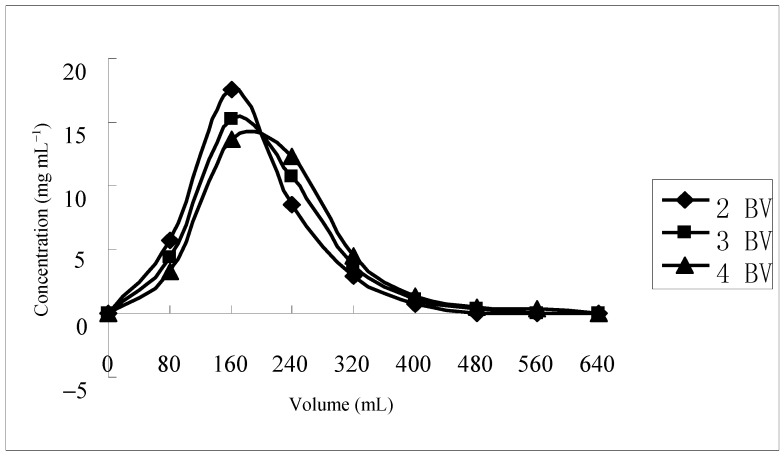
The effects of eluate flow rate on ratio of desorption with the ethanol-water (40%, *v*/*v*) solution in dynamic adsorption.

The flow rates investigated in this test were 2, 3 and 4 BV**·**h^−1^, and an ethanol-water solution (40%, *v*/*v*) was used to elute total flavonoids, with the results shown in Figure 4. Although the elution volume varied little with increasing the flow rate, total flavonoids were totally desorbed with 5 BV, 6 BV and 7 BV of elution volume at the flow rate of 2, 3 and 4 BV**·**h^−1^, respectively. Thus, 2 BV**·**h^−1^ was selected as the proper desorption flow rate in consideration of the lower volume consumption and high efficiency in the dynamic desorption experiments. 

### 2.5. Gradient Elution in Dynamic Adsorption and Desorption 

On basis of the above experiments, gradient elution test was conducted under the following conditions: The concentration of the crude flavonoid extracts in sample solutions was 20 mg**·**mL^−1^ for the adsorption process; in desorption process, the flow rate was 2 BV**·**h^−1^ and the eluents were ethanol-water solutions (5%, 10%, 15%, 20%, 25%, 30%, 35%, 40%, 60%, *v*/*v*). Gradient elution has separation effects for different kinds of chemical constituents, TLC can be used to detect the separation effects according the spot color, and thus all the eluates were analyzed by TLC. The volumes of eluents needed in the gradient elution and TLC results of eluates were shown in Table 2. As shown in Table 2, the spots from the eluates of 10%–30% aqueous ethanol (*v*/*v*) were yellow, the spots from the eluates of water and 5% aqueous ethanol (*v*/*v*) were purple or brown, and the spots from the eluates of above 30% aqueous ethanol (*v*/*v*) were brown. In our previous study, we found that purple spots were from chlorogenic acid and its derivatives, the brown spots were from terpenoid compounds, and only yellow spots were from flavonoid compounds. Those indicated that the elution with water and 5% (*v*/*v*) aqueous ethanol can remove the chlorogenic acid and part of its derivatives from the crude flavonoid extracts, then flavonoid compounds can be obtained by eluting with 10%–30% (*v*/*v*) aqueous ethanol, and last the elution solvents above 30% (*v*/*v*) aqueous ethanol were used to clean the resin. In conclusion, the optimal gradient elution is to elute successively with 5 BV of water, 5 BV of 5% (*v*/*v*) aqueous ethanol solution and 5 BV of 30% (*v*/*v*) aqueous ethanol solution, and the total flavonoids were obtained from the eluate of 30% (*v*/*v*) aqueous ethanol solution.

### 2.6. Preparation Method Validation 

On basis of the above experiments, the appropriate preparation and purification process of total flavonoids from crude flavonoid extracts of *S. austriaca* on D4020 resin was as follows: the pretreated D4020 resin column was loaded with the crude flavonoid extract solution at the initial concentration of 20 mg**·**mL^−1^. After reaching adsorptive saturation, the column was eluted successively with 5 BV of water, 5 BV of 5% (*v*/*v*) aqueous ethanol and 5 BV of 30% (*v*/*v*) aqueous ethanol, and the elute flow rate is 2 BV**·**h^−1^. The total flavonoids were obtained from the eluate of 30% (*v*/*v*) aqueous ethanol solution by vacuum distillation recovery. 

Six total flavonoids samples were prepared from the same sample of the crude flavonoid extracts of *S. austriaca* by this process and their contents and recovery yields were determined by the ultraviolet-visible spectrophotometry method (Table 3). As shown in Table 3, the total contents of total flavonoids in final products were more than 90%, which had been improved about 150% after the preparation and purification process procedure, compared to the content of total flavonoids in the crude flavonoid extracts (62.1%). And the RSD (%) was 1.85, indicating that this method is stable and feasible.

### 2.7. HPLC Analysis of the Final Products and Crude Flavonoid Extracts of Scorzonera austriaca 

**Figure 5:**
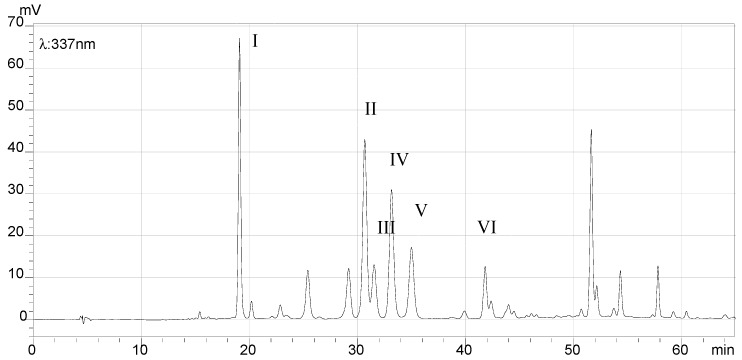
HPLC chromatogram of the crude flavonoid extracts (λ = 337 nm). The peaks were identified as chlorogenic acid (I), 2″-*O*-β-d-xylopyranosyl isoorientin (II), 6-*C*-α-l-arabipyranosyl orientin (III), orientin (IV), isoorientin (V), and vitexin (VI).

**Figure 6:**
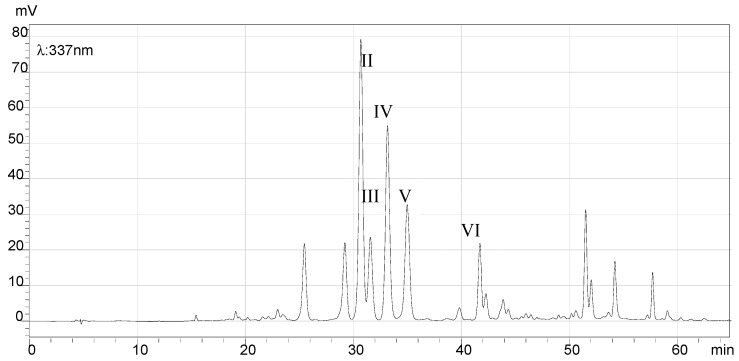
HPLC chromatogram of the final product (λ = 337 nm). Comparing with Figure 5, the peak of chlorogenic acid (I) did not appeared.

**Figure 7:**
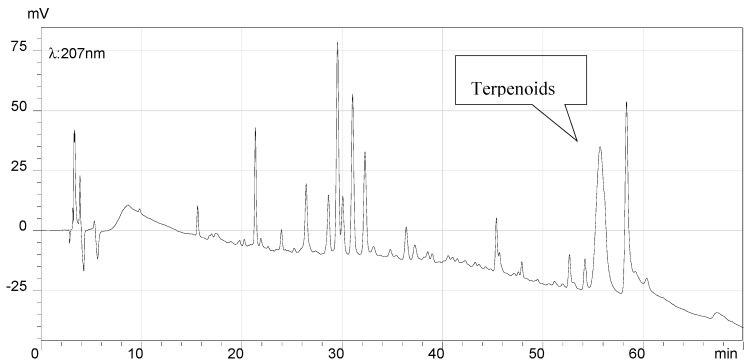
HPLC chromatogram of crude flavonoid extracts (λ = 207 nm). The broad peak with retention time of 55.47 min was terpenoids.

**Figure 8:**
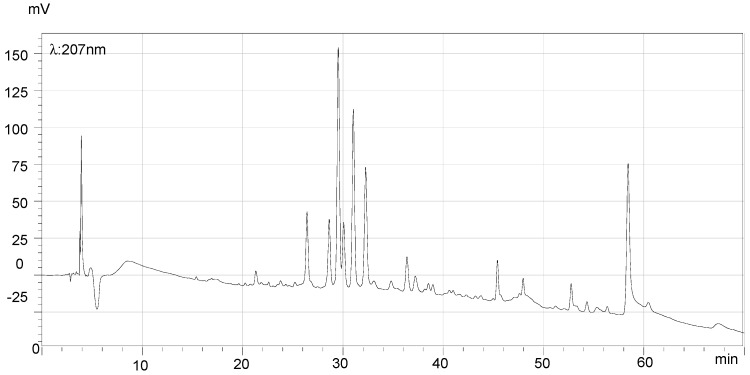
HPLC chromatogram of final products (λ = 207 nm). Comparing with Figure 7, the broad peak with a retention time of 55.47 min disappeared.

Figure 5, Figure 6, Figure 7 and Figure 8 illustrate the HPLC chromatograms of the final products and crude flavonoid extracts of *S. austriaca* and the structural assignments of the six peaks are given in Table 4. Structural elucidations were done by LC-ESI-MS analysis and by using internal standard methods with available reference standards. As shown in Figure 5, six peaks with retention times of 19.08, 30.67, 31.54, 33.15, 34.99 and 41.81 min in the HPLC chromatogram of crude flavonoid extracts were identified as chlorogenic acid [17], 2″-*O*-β-d-xylopyranosyl isoorientin [18], 6-*C*-α-l-arabipyranosyl orientin [18], orientin [19], isoorientin [19] and vitexin [18], and the content of orientin was 9.5%. However, in the HPLC chromatogram of the final products (Figure 6), the chlorogenic acid peak with a retention time of 19.08 min disappeared, the peak area of all other peaks identified increased, and the content of orientin increased to 13.4%, which suggested that this preparation and purification process can remove the chlorogenic acid and enhance the content of total flavonoids. In the HPLC chromatograms recorded at detection wavelength 207 nm (Figure 7 and Figure 8), the terpenoid peaks disappeared in the HPLC chromatogram of the final products, which indicated that this preparation and purification process can also remove terpenoids. 

## 3. Experimental Section 

### 3.1. Materials 

*Scorzonera austriaca* herbs were collected in Siping District in Jilin Province, China, and identified by Prof. Jing-min Zhang of School of Pharmaceutical Sciences, Jilin University, Changchun, China. Distilled water was purchased from Hangzhou Wahaha Group Co., Ltd. (Hongzhou, China). Acetonitrile of chromatographic grade for HPLC was purchased from Fisher Scientific (Fair Lawn, NJ, USA). Chlorogenic acid was purchased from the Chinese National Institute for the Control of Pharmaceutical and Biological Products (Beijing, China). 2″-*O*-β-d-Xylopyranosyl isoorientin, 6-*C*-α-l-arabipyranosyl orientin, orientin, isoorientin, and vitexin were prepared in our laboratory [18,19]. Resins were purchased from Chemical Industry of Nankai University (Tianjing, China). Other chemicals and reagents were analytical grade from Beijing Chemical Works (Beijing, China). The reference compound, orientin, was accurately weighed and dissolved in a 60% aqueous ethanol solution (*v*/*v*) to prepare a standard stock solution. Working standard solutions for the calibration curves were prepared by diluting the standard solution with 60% aqueous ethanol solution (*v*/*v*) to the appropriate concentrations. All solvents and solutions were filtered through a nylon membrane (0.45 μm pore size) before HPLC.

### 3.2. Pretreatment of Adsorbents 

Macroporous resins including D101, D4020, NKA-9, X-5 and AB-8 were soaked in 95% aqueous ethanol solution (*v*/*v*) for 24 h, loaded on chromatographic columns, first washed by 95% aqueous ethanol solution (*v*/*v*) until the eluate is no longer cloudy after adding water, and finally washed by distilled water thoroughly. Then the resins were soaked in 5% HCl for 4 h and washed by distilled water to neutral, soaked in 5% NaOH for 4 h and washed by distilled water to neutral, and finally dried at 70 °C in a drying oven. Prior to use in the adsorption experiments, the dried resins were immersed in ethanol for 12 h and subsequently the ethanol was replaced by distilled water through washing. 

### 3.3. Preparation of the Crude Flavonoid Extracts from Scorzonera austriaca Herbs

According to the method reported in literature [18], 2 kg of air-dried whole *S. austriaca* herbs were extracted twice with 20 L of 70% aqueous ethanol solution (*v*/*v*) at room temperature. The extraction solution was concentrated under reduced pressure to remove ethanol, and the water concentrate was filtered and then passed through a D101 polyporous resin column eluting successively with water and 60% aqueous ethanol solution (*v*/*v*). The crude flavonoid extracts were obtained from 60% aqueous ethanol eluate by vacuum distillation recovery and used for the next experiments. The content of total flavonoids in the crude flavonoid extracts was 62.1% by using the following ultraviolet-visible spectrophotometry method and the content of orientin in the crude flavonoid extracts is 9.5% by using the following HPLC method.

### 3.4. Ultraviolet-Visible Spectrophotometry Method for Determination of Flavonoid Compounds 

The concentration of total flavonoids was determined according to the following method. Certain amount of standard solution or sample solutions, 3.0 mL of 1% AlCl_3_-MeOH and 3.0 mL of HAc-NaAc buffer solution (pH = 5) were transferred to a 10-mL volumetric flask and diluted with 60% aqueous ethanol solution (*v*/*v*) to volume, and after one hour of coloration the absorbance (A) was measured using a UV-VIS spectrophotometer at 390 nm in duplicate. A calibration curve, A = 19.676 X + 0.004 (R^2^ = 0.9999, linear range: 0.001–0.0416 mg**·**mL^−1^), was established by plotting the absorbance (A) of the reference compound, orientin, against the concentrations (X) of the standard solutions, and then the amounts of total flavonoids expressed as orientin equivalents in the samples were then calculated using the calibration curve.

### 3.5. HPLC/ESI/MS Analysis of Total Flavonoids 

HPLC analysis of total flavonoids was carried out on a LC-10AT VP Plus system (Shimadzu, Kyoto, Japan). Analysis was performed on a Phenomenex Gemini ODS column (4.6 mm × 250 mm, 5 μm). The following gradient elution procedure was used for the analysis of the samples: The mobile phase components were acetonitrile (A) and 0.1% formic acid solution in water (B) and the linear gradient program was 3%–14% A from 0–7 min, 14%–17% A from 7–30 min, 17%–35 % A from 30–60 min, and 35%–100 % A from 60–65 min, the mobile phase flow rate was 0.8 mL**·**min^−1^, the column temperature was kept at 25 °C, the injection volume was 10 μL, and the eluate was monitored at 337 or 207 nm. The mass spectra were obtained on a Bruker microOTOF-Q II mass spectrometer (Bruker Corporation, Bremen, Germany). Acquisition Parameters: Source Type: ESI; Focus: Active; Scan Begin: 50 *m*/*z*; Scan End: 3000 *m*/*z*; Ion Polarity: Positive; Capillary: 4500 V; End Plate Offset: −500 V; Collision Cell RF: 150.0 Vpp; Nebulizer: 0.8 Bar; Dry Heater: 180 °C; Dry Gas: 8.0 L min^−1^; Divert Valve: Waste.

Identification of compounds was achieved by comparing their mass data with those reported in the literature and by using internal standard methods with available reference standards. The content of orientin was calculated with the regression equation, Y = 2.379601 × 10^7^ X − 69815.1 (Y, peak area; X, concentrations of the standard solutions; R^2^ = 0.9998; linear range, 0.04–0.28 μg**·**μL^−1^) from the standard curve by using an external standard method. The results were averages of triplicate determinations.

### 3.6. Static Adsorption and Desorption Tests 

The static adsorption and desorption tests of total flavonoids on macroporous resins were performed as follows: 1 g of pretreated resin together with 50 mL of 1.40 mg·mL^−1^ crude flavonoid extract solution was added into a flask, shaken (130 rpm) for 24 h at 25 °C. After adsorption, the resin was desorbed with 50 mL of 95% aqueous ethanol solution (*v*/*v*), and shaken (130 rpm) for 24 h at 25 °C. The respective concentrations of total flavonoids in the sample solution after adsorption of a certain time were monitored at equal time intervals till equilibration to get adsorption kinetic curves. The selectivity of resins was based on the capacities of adsorption and desorption, the ratio of desorption and the rate of adsorption. The tests for the maximum adsorption capacity on the selected resin were conducted by contacting 50 mL sample solutions at different concentrations with 1 g of pre-weighed resins. The effects of ethanol concentrations on the ratio of desorption were carried out by desorbing successively with aqueous ethanol solutions of different ethanol concentrations. All the processes were repeated for three times.

### 3.7. Dynamic Adsorption and Desorption Tests 

Dynamic adsorption and desorption experiments for total flavonoids were carried out on glass columns (2.5 mm × 300 mm) wet-packed with 70 g (wet resin) of the selected hydrated resin. The bed volume (BV) of resin was 100 mL. After reaching adsorptive saturation, the column was first washed by distilled water with 5 BV, and then eluted by aqueous ethanol solutions. The gradient elution tests were taken as follows: the column was first washed by distilled water with 5 BV, and then eluted by different aqueous ethanol solutions, in which the eluate was collected every 20 mL and the elution process of each ethanol concentration was carried out until the eluate was colorless. The eluates were analyzed by TLC (plates: glass precoated silica gel GF_254_ plates; developing solvent: CHCl_3_-MeOH-EtOAc-H_2_O, 2:2:4:1, *v*/*v*, lower layer; detection method: spraying with 10% H_2_SO_4_ in 95% ethanol followed by heating and leaving overnight), merged and then concentrated to dryness under vacuum. The effects of two variables, ethanol/water ratios and elute flow rates, on desorption properties of the selected resin were also studied. All the results were averages of triplicate determinations.

## 4. Conclusions 

A method for the preparative separation and purification of total flavonoids from the crude flavonoid extracts of *S. austriaca* with macroporous resin has been successfully developed in this study. The static adsorption and desorption characteristics of five widely used macroporous resins for total flavonoids in the crude flavonoid extracts of *S. austriaca* were evaluated. Among the resins investigated, D4020 was selected as a suitable resin for total flavonoid separation because of its comparatively high adsorption capacity and ratio of desorption. Parameters of the initial concentration of the crude flavonoid extracts, elution solvent concentration and elute flow rate were optimized with the static/dynamic adsorption and desorption tests. The separation effects of the gradient elution had been also evaluated according to the color of the spots in TLC, and thus the optimal gradient elution were selected. In conclusion, the appropriate preparation and purification process of total flavonoids from crude flavonoid extracts of *S. austriaca* on D4020 resin was as follows: the pretreated D4020 resin column was loaded with the crude flavonoid extract solution at the initial concentration of 20 mg·mL^−1^, and after reaching adsorptive saturation, the column was eluted successively with 5 BV of water, 5 BV of 5% (*v*/*v*) aqueous ethanol and 5 BV of 30% (*v*/*v*) aqueous ethanol at the elute flow rate of 2 BV·h^−1^. The total flavonoids were obtained from the eluate of 30% (*v*/*v*) aqueous ethanol by vacuum distillation recovery.

Using the D4020 resin at the optimal conditions, the total content of total flavonoids in the final product was 93.5%, which represents an improvement by about 150% after the preparative separation and purification procedure. The HPLC chromatograms recorded at the detection wave lengths of 337 and 207 nm demonstrated that this preparation and purification process can remove chlorogenic acid and terpenoids. Validation experiments showed that the method was stable and feasible.

This method has the advantages of low cost, high efficiency, procedural simplicity and ease in upscaling compared to the existing methods for total flavonoid purification such as high speed counter-current chromatography, solvent extraction, supercritical carbon dioxide extraction, aqueous two-phase flotation, and could be referenced for the preparative separation and purification of total flavonoids from *S. austriaca* in industry.

## Figures and Tables

**Table 1:** Adsorption and desorption properties of five macroporous resins for total flavonoids in the crude flavonoid extracts of *Scorzonera austriaca*.

Trade Name	Q_a_^a^ (mg·g^−1^)	Q_d_^a^ (mg·g^−1^)	R_d_^a^ (%)
D101	26.4 ± 2.6	20.7 ± 2.1	78.4 ± 2.3
D4020	38.7 ± 2.1	34.8 ± 3.0	90.1 ± 2.6
NKA-9	28.1 ± 3.5	25.1 ± 3.1	89.4 ± 3.3
X-5	22.8 ± 1.7	15.6 ± 1.9	68.5 ± 2.5
AB-8	26.2 ± 2.3	23.0 ± 2.6	87.7 ± 4.3

**^a^** Means of three determinations ± SD.

**Table 2:** The volumes of eluents needed in the gradient elution and TLC results of eluates.

Eeluate	Water	5%	10%	15%	20%	25%	30%	35%	40%	60%
Volume needed (BV)	5	5	5	4	5	4	5	3	2	2
Colour of TLC spots	purple	purple, brown	yellow	yellow	yellow	yellow	yellow	brown	brown	brown

**Table 3:** Verified results of preparation and purification tests by D4020 macroporous resin.

No.	1	2	3	4	5	6	mean	RSD/%
^a^ Recovery yield (%)	63.40	65.55	64.46	62.50	61.72	63.83	63.58	1.37
Content (%)	93.68	90.95	92.54	94.28	96.50	93.18	93.52	1.85

^a^ Recovery yield = weight of final product/weight of crude flavonoid extracts × 100.

**Table 4:** Peak assignment for the compounds in the final product and the crude flavonoid extracts of *Scorzonera austriaca*.

Peak No.	Retention Time (min)	HRMS (*m*/*z*) [M + H]^+^	MS (*m*/*z*) Caculated	Structural Identification	Reference
I	19.08	355.1027	355.1024	Chlorogenic acid	[17]
II	30.67	581.1527	581.1501	2″-*O*-β-d-xylopyranosyl isoorientin	[18]
III	31.54	565.1580	565.1552	6-*C*-α-l-arabipyranosyl orientin	[18]
IV	33.15	449.1089	449.1078	Orientin	[19]
V	34.99	449.1093	449.1078	Isoorientin	[19]
VI	41.81	433.1156	433.1129	Vitexin	[18]

## References

[1] The Editorial Board of Zhong Hua Ben Cao of State Administration of Traditional Chinese Medicine of the People’s Republic of China (1999). Zhong Hua Ben Cao.

[2] Zhang T.W., Xie Y., Zhang Z., Wang G.S. (2015). Study on hepatoprotective effects of total flavonoids in *Scorzonera austriaca* Wild *in vivo* and *in vitro*. Chin. J. Biochem. Pharm..

[3] Wang G.S., Yang X.H., Zhou X.P. (2013). Aplication of the extract of *Scorzonera austriaca* in preparing medicine to treat hepatitis. China Patent.

[4] Xie Y., Wang J., Geng Y.M., Qu Y.F., Zhang Z., Wang G.S. (2015). Experimental studies on inhibitory effects of total flavonoids in *Scorzonera austriaca* Wild on hepatitis B virus *in vitro*. Chin. J. Biochem. Pharm..

[5] Xiao X.H., Si X.X., Tong X., Li G.K. (2011). Preparation of flavonoids and diarylheptanoid from *Alpinia katsumadai Hayata* by microwave-assisted extraction and high-speed counter-current chromatography. Sep. Purif. Technol..

[6] Qi Y.Y., Sun A.L., Liu R.M., Meng Z.L., Xie H.Y. (2007). Isolation and purification of flavonoid and isoflavonoid compounds from the pericarp of *Sophora japonica* L. by adsorption chromatography on 12% cross-linked agarose gel media. J. Chromatogr. A.

[7] Luan N., Li D. (2010). Study on supercritical CO_2_ extraction of flavonoids from *Cynomorium songaricum*. J. Chin. Med. Mater..

[8] Chang L., Wei Y., Bi P.Y., Shao Q. (2014). Recovery of liquiritin and glycyrrhizic acid from *Glycyrrhiza uralensis Fisch* by aqueous two-phase flotation and multi-stage preparative high performance liquid chromatography. Sep. Purif. Technol..

[9] Song X.L., Li J.H., Wang J.T., Chen L.X. (2009). Quercetin molecularly imprinted polymers: Preparation, recognition characteristics and properties as sorbent for solid-phase extraction. Talanta.

[10] Lv L.S., Tang J., Ho C.T. (2008). Selection and optimization of macroporous resin for separation of stilbene glycoside from *Polygonum multiflorum* Thunb. J. Chem. Technol. Biotechnol..

[11] Li C., Zheng Y.Y., Wang X.F., Feng S.L., Di D.L. (2011). Simultaneous separation and purification of flavonoids and oleuropein from *Olea europaea* L. (olive) leaves using macroporous resin. J. Sci. Food Agric.

[12] Lin L.Z., Zhao H.F., Dong Y., Yang B., Zhao M.M. (2012). Macroporous resin purification behavior of phenolics and rosmarinic acid from *Rabdosia serra* (Maxim.) Hara leaf. Food Chem..

[13] Lin X.Q., Wu J.L., Fan J.S., Qian W.B., Zhou X.Q., Qian C., Jin X.H., Wang L.L., Bai J.X., Ying H.J. (2012). Adsorption of butanol from aqueous solution onto a new type of macroporous adsorption resin: studies of adsorption isotherms and kinetics simulation. J. Chem. Technol. Biotechnol..

[14] Liu W., Zhang S., Zu Y.G., Fu Y.J., Ma W., Zhang D.F., Kong Y., Li X.J. (2010). Preliminary enrichment and separation of genistein and apigenin from extracts of pigeon pea roots by macroporous resins. Bioresour. Technol..

[15] Zhang M., Yang H.H., Zhang H.Y., Wang Y.R., Hu P. (2011). Development of a process for separation of mogroside V from *Siraitia grosvenorii* by macroporous resins. Molecules.

[16] He Z.Y., Xia W.S. (2008). Preparative separation and purification of phenolic compounds from *Canarium album* L. by macroporous resins. J. Sci. Food Agric..

[17] Li J., Yu D.Q. (2011). Chemical constituents from herbs of *Erigeron breviscapus*. Chin. J. Chin. Mater. Med..

[18] Liu X., Zhang Z.L., Wang Z., He L., Peng C., Wang G.S. (2012). Isolation and structure identification of flavonoid glycosides from *Scorzonera austriaca* Wild. J. Jilin Univ. Med. Ed..

[19] Li Q.M., Wang G.S. (2010). Isolation and structure identification of flavonoid glycosides from *Scorzonera ruprechtiana* Lipsch et Krasch. J. Jilin Univ. Med. Ed..

